# Commentary on the Risk Assessment of Lead by the Food Safety Commission of Japan

**DOI:** 10.14252/foodsafetyfscj.D-22-00007

**Published:** 2022-09-23

**Authors:** Fumi Irie

**Affiliations:** Department of Health Care Administration and Management, Graduate School of Medical Sciences, Kyushu University, 3-1-1 Maidashi, Higashi-ku, Fukuoka 812-8582, Japan

**Keywords:** blood lead level, human biomonitoring, lead, neurodevelopmental effects, renal effects, risk assessment.

## Abstract

This article describes in detail the process of and the basis for the risk assessment of lead, started as a self-tasking assessment in April 2008 and finalized in June 2021 by the Food Safety Commission of Japan (FSCJ). Discussion points addressed in the working group set under the FSCJ in April 2019 are also presented in this commentary. To reflect the overall exposure to lead from various sources, blood lead level (BLL) was used as the basic metric for the assessment. For the evaluation of effects on human health, the approach of overall weight of evidence was taken, rather than selecting one critical endpoint, in consideration of the uncertainties inherent to epidemiological studies, particularly those examining the effects associated with low-level lead exposure. The overall evidence compiled for the assessment suggested that BLLs in the range of 1–2 μg/dL might be associated with some effects on human health. The representative value of BLL for the entire population was difficult to obtain due to the lack of a national population-based survey in Japan. Instead, the current average BLL of the Japanese population was estimated based on recent studies conducted in Japan. The estimated average exposure level was below or equal to 1 μg/dL and close to the levels at which some effects on human health might occur, as suggested by epidemiological studies. Hence, the continued enforcement of measures to reduce lead exposure is indispensable. Furthermore, a national human biomonitoring program to continuously assess the exposure status of the Japanese population, which can be ultimately used for assuring the effectiveness of control measures, is needed.

## 1. Introduction

In April 2008, the Food Safety Commission of Japan (FSCJ) decided to conduct a risk assessment of lead within its framework for self-tasking assessment^*^. Prior to this, FSCJ was requested to conduct a risk assessment of lead by the Ministry of Health, Labour and Welfare (MHLW) in 2003 as part of the revision process of the standards for bottled drinking water. MHLW also informed the FSCJ of its intent to strengthen the regulation of lead in apparatus/container and packaging (ACP) in 2007. Considering the necessity of aligning the maximum levels of lead in foodstuffs set by the MHLW with international guideline levels, as well as the wide distributions of lead across various food categories, the FSCJ deemed it appropriate to evaluate the risks of the presence of lead in food to human health in a comprehensive way, rather than to evaluate only the risks of lead exposure from beverages and ACP.

A working group (WG), to which experts from several committees of the FSCJ participated, was established in May 2008 to conduct a comprehensive risk assessment of lead in food. The evaluation of the risk related to lead contamination through ACP was also within the scope of the WG. Over ten meetings (June 2008–March 2010), various data relevant to risk assessment of lead, from results of epidemiological studies to experimental data on possible modes of action, were reviewed by the WG. The WG issued an interim report on the health effects related to lead exposure in March 2012. A blood lead level (BLL) of 4 μg/dL was proposed as a level below which no adverse effects would occur in infants and children. The BLL of 4 μg/dL was recognized as applicable to pregnant women and women of childbearing age, and a BLL of 10 μg/dL was proposed for other adults. These proposed levels were derived from critical appraisal and dose-response analyses of epidemiological studies, focusing on the neurotoxic effects of lead^1^^,^^2^^,^^3^^)^. This interim report also included an exposure assessment based on total diet studies over the past decades in Japan as well as reported data on BLLs of children and adult women from two studies recently conducted in Japan.

Judging from the available evidence at that time, the WG concluded that BLLs of Japanese were maintained below the levels of concern, i.e., the BLL of 4 μg/dL for infants and children and the BLL of 10 μg/dL for adults, derived from the assessment of lead-induced adverse effects^1^^,^^2^^,^^3^^)^. This report by the WG was, however, not finalized as a risk assessment report by the FSCJ due to the difficulty of the conversion of levels of concern expressed as BLLs into dietary exposure values. Existing estimation models for the relationship between BLLs and dietary lead intake were developed during a period of relatively high lead levels and thus might not be applicable to the evaluation of the current situation. Data to determine representative values of non-dietary exposure in Japanese individuals were also unavailable. These issues precluded the use of the existing models. The assessment was halted at the proposition of BLLs below which no adverse effects would occur. No tolerable intake level of lead from food was thus proposed in the interim report.

The FSCJ established a new WG on lead to conduct a risk assessment with updated data in April 2019. Over eight meetings (May 2019–April 2021) of the new WG, discussions focused on the possible identification of a BLL below which no adverse health effects would occur and on the conversion of BBLs into dietary lead exposure values. The final risk assessment report was adopted at the 822nd plenary session of the FSCJ, on June 29, 2021.

This article describes the major discussion points and limitations revealed by this risk assessment of lead, which required more time to conduct than does the usual due to the difficulties related to the evaluation of human health effects of low-dose exposure and the consideration of different routes of exposure other than ingestion. Future perspectives derived from the experience of this risk assessment are also presented at the end of the article.

## 2. Evaluation of Human Health Effects

The WG reviewed the literature on human health effects of lead exposure, focusing primarily on recent scientific evidence of the effects associated with BLLs lower than 4 μg/dL for infants and children and 10 μg/dL for adults.

### 2.1. Effects on Intelligence Quotient (IQ) in Children

The interim report prepared by the former WG and issued in 2012 identified neurodevelopmental effects in children as a critical endpoint^1^^,^^2^^)^. However, the principal data used for the assessment at that time were from Western countries, which differ from Japan in both ethnic and socioeconomic aspects.

For the risk assessment restarted in 2019, the new WG carefully evaluated the results of a recently published study using data of 289 Japanese children from a prospective birth cohort, that is, the Tohoku Study of Child Development. This study demonstrated that BLLs in the range of 1–2 μg/dL at the age of 12 years were associated with a decrease in Full Scale Intelligence Quotient (FSIQ) among boys^4^^)^. A birth cohort study conducted in Taiwan also reported an inverse relationship between BLLs (around 2 μg/dL) at the age of 5-6 years and IQ at the age of 8-9 years^5^^)^. In addition, the WG recognized the dose-response assessment conducted by the European Food Safety Authority (EFSA) using IQ deficits in children as a critical endpoint, based on a pooled analysis of 1333 children^6^^)^. This dose-response assessment resulted in a benchmark dose lower confidence limit (BMDL) of BLL 1.2 μg/dL for a benchmark response of 1%, i.e., 1 point decrease in FSIQ^7^^)^.

Given the accumulated results of epidemiological studies along with mechanistic data and evidence from animal studies, the WG considered that the level of confidence in the neurodevelopmental effects of low-level lead exposure was high. Moreover, recognizing the fact that prospective studies published since 2012, including the one conducted in Japan, suggested associations between decrements in IQ and BLLs as low as 1–2 μg/dL, the BLL of 4 μg/dL proposed as a “threshold” level for infants and children in the interim report was no longer considered appropriate.

Nevertheless, several uncertainties must be considered. Factors other than lead exposure, such as socioeconomic status and co-exposure to other chemicals, can affect intellectual and behavioral development, but not all factors can be accounted for in epidemiological studies. In addition, concerns were expressed during the WG meetings over the fact that the instruments for assessing neurobehavioral development, including IQ tests, are not specific tools for neurotoxicity assessment of environmental hazards, and substantial inter- and intra-individual variations exist. Furthermore, the WG noted that a decrease in IQ score that can be regarded as substantial at the population level might have only a small effect at the individual level. Therefore, the WG concluded that it was not possible to identify a BLL below which no adverse effects on neurodevelopment would occur in infants and children.

### 2.2 Behavioral Effects in Children

With regard to behavioral effects, three cross-sectional studies examining data from the National Health and Nutrition Examination Survey (NHANES) between 2001 and 2004 reported increased odds ratios of conduct disorder, attention-deficit/hyperactivity disorder, and learning disabilities among children with BLLs in the range of 1–2 μg/dL compared with those with BLLs below 1 μg/dL^8^^,^^9^^,^^10^^)^. In contrast, no effect was observed in a group of children with BLLs of 2–5 μg/dL compared to a reference group of BLLs of 0–2 μg/dL in a longitudinal prospective study examining the impact of lead exposure at 30 months on educational and behavioral outcomes at the age of 7–8 years using data from a birth cohort in the United Kingdom. In this study, BLLs of 5–10 μg/dL were associated with reduced reading and writing scores^11^^)^.

Considering the inconsistency in the results of epidemiological studies, the WG concluded that the relationship between behavioral and learning problems and BLLs as low as 1–2 μg/dL was less clear than that between deleterious effects on IQ and BLLs within the same range. Various uncertainties were pointed out, including different diagnoses of developmental disorders, cross-sectional design of NHANES, and interpretation of results of studies conducted in socio-economically and ethnically different contexts from those of Japan.

### 2.3. Renal Effects in Adults

As for effects associated with low-level lead exposure in adults, renal effects have been intensively studied in the past decades. Two cross-sectional studies using data from the NHANES around the year 2000 reported significantly high odds ratios of chronic kidney disease (CKD), defined as a glomerular filtration rate (GFR) below 60 mL/1.73 m^2^/min, in groups of subjects with BLLs of approximately 2 μg/dL or higher compared with those with BLLs of approximately 1 μg/dL or lower^12^^,^^13^^)^. However, in a more recently published cohort study in Sweden in which the mean BLL of study subjects was 2.5 μg/dL, the hazard ratio for CKD of the highest quartile (3.30 to 25.8 μg/dL) was not significantly higher than the lowest quartile (0.15 to 1.85 μg/dL)^14^^)^.

In addition to the inconsistency in the results of epidemiological studies, the WG noted that experimental evidence supporting the causal relationship between reduced GFR and lead exposure was not as strong as that for decreased IQ score. Furthermore, concerns were raised during discussions among the WG members regarding the following points: since nephrotoxicity is not a lead-specific endpoint, the effect of co-exposure to other chemicals, in particular cadmium, needs to be taken into account; aging, diabetes mellitus, and hypertension have already been identified as risk factors for impaired renal function, and therefore the additive effect of low-level lead exposure should be carefully examined among those who have these risk factors; since urinary excretion is a primary route of lead excretion, the possibility of reverse causation cannot be excluded in studies of cross-sectional design; the fact that the value of GFR is estimated from serum creatinine concentration, which is substantially influenced by age and the volume of body muscles, can be a source of uncertainty.

### 2.4. Cardiovascular Effects in Adults

The cardiovascular effects of low chronic lead exposure in the general population have been studied for decades. In particular, increased blood pressure is a well-known endpoint. However, recent studies have reported conflicting results regarding the association between BLLs below 10 μg/dL and effects on blood pressure; some studies reported no significant association at BLLs around 3 μg/dL^15^^,^^16^^)^, whereas others found increases in systolic or diastolic blood pressure at BLLs in the range of 1–2 μg/dL^17^^-^^20^^)^. An explanation for this inconsistency would be the intra-individual variability in blood pressure. Another possible reason for the inconsistency could be differences in study populations, as significant associations were only found in susceptible populations, such as pregnant women and certain ethnic groups^17^^-^^19^^)^. Moreover, during the WG meetings, several views were expressed on whether an increase in blood pressure to a certain extent was a reversible phenomenon; as such increased blood pressure was not an ultimate clinical outcome, but rather a risk factor for adverse outcomes such as stroke and coronary heart disease. Therefore, the clinical significance of elevated blood pressure related to low-level lead exposure was considered limited.

Cardiovascular disease (CVD) is an important endpoint from a public health perspective, as CVD remains one of the leading causes of death in Japan. Moreover, CKD, which has been suggested to be one of the major clinical outcomes in the assessment of lead toxicity in adults, is an important risk factor for CVD. Hence, CVD can be considered the ultimate outcome for examining the health effects of lead exposure.

Two follow-up studies based on the NHANES database reported increases in cardiovascular mortality associated with low-level lead exposure in the general population: the mean BLL reported in one study was 1.73 μg/dL among subjects of the NHANES between 1999 and 2010, and the median BLL reported in the other study was 1.62 μg/dL among subjects of the NHANES between 1999 and 2012^21^^,^^22^^)^. However, since both studies used data from the NHANES during overlapping periods, methodological limitations should be considered. Additionally, it is well established that multiple factors contribute to the development of CVD, including aging, hypertension, diabetes mellitus, dyslipidemia, and smoking. The extent to which the variance in cardiovascular mortality is attributable to lead exposure needs to be carefully examined.

### 2.5. Neurotoxicity in Adults

Neurotoxicity of lead in adults was well documented in the field of occupational medicine in the past. Symptoms of lead poisoning included encephalopathy and peripheral neuropathy, suggesting that the nervous system is the target organ of lead. In recent decades, subclinical effects of chronic lead exposure, such as decreased peripheral nerve conduction velocity and deficits in neuropsychological and neurobehavioral tests, have been documented at lower exposure levels than the levels of lead poisoning.

The interim report in 2012 deduced that a BLL of 10 μg/dL was a critical level for adults based on BMD analyses of findings on neurophysiological endpoints, including maximal motor nerve conduction velocity and postural balance, among occupationally exposed workers^3^^)^. However, recent literature has suggested that renal and cardiovascular effects of lead occur at BLLs below 10 μg/dL. Consequently, the level of 10 μg/dL was not retained in the final risk assessment report of the FSCJ.

Two studies using the NHANES database reported associations between hearing loss and BLLs in the range of 1–2 μg/dL^23^^,^^24^^)^. However, considering the limitations related to the cross-sectional design of the NHANES, the WG concluded that further evidence is needed to confirm the ototoxic effects of lead exposure at such low levels.

### 2.6. Summary of Observations in Humans

Judging from the overall body of evidence provided by previous epidemiological studies on the effects of chronic lead exposure, the WG recognized the possibility that some effects on human health, such as neurodevelopmental effects in children and renal effects in adults, can occur at BLLs as low as 1–2 μg/dL. There seemed to be no difference in the BLLs at which some effects were observed between children and adults. Therefore, no susceptible subpopulation was defined in the current risk assessment.

The WG also recognized uncertainties related to the assessment of human health effects at such low levels. First, uncertainties were associated with the endpoints to be used in the hazard characterization of lead. For endpoints other than neurotoxicity, findings in epidemiological studies on the effects of low-level lead exposure were inconsistent across studies. Often, significant associations were found only in cross-sectional studies but not in cohort studies. In addition, experimental evidence providing information on the mechanisms of action of lead at low levels was limited for most endpoints. Hence, it was difficult to conclude the presence of a causal relationship between the observed effects and low-level lead exposure. Furthermore, the significance of the observed changes from the clinical or public-health perspective for endpoints such as an increase in blood pressure and a decrease in GFR was not as evident as that of a decrease in IQ score. Second, the effect of lead exposure per se, especially at BLLs as low as 1–2 μg/dL, was difficult to estimate because confounding by known risk factors, such as age, hypertension, diabetes mellitus, and smoking, could be more important than low-level lead exposure for decreased GFR and cardiovascular mortality. The possibility of confounding could not be completely excluded because of methodological limitations inherent to epidemiological studies; therefore, the true effect of low-level lead exposure remained unmeasurable. Third, the variability in measurement protocols of endpoints such as GFR and blood pressure, as well as in diagnostic methods for neurodevelopmental disorders, could hinder an appropriate assessment of the dose-response relationship. Lastly, the use of epidemiological data from Western countries due to the paucity of data in Japan could be a source of uncertainty, as previous studies reported ethno-racial differences for certain endpoints, such as hypertension^18^^)^ and CKD^12^^)^.

Because of these uncertainties, the WG considered it difficult to conduct a precise dose-response assessment for any individual endpoint based on the currently available epidemiological data. Therefore, the WG decided to take the overall weight of the evidence approach by comprehensively evaluating the findings across endpoints of concern. Based on a careful evaluation, the WG concluded that BLLs in the range of 1–2 μg/dL could have some effects on human health, and that the current body of evidence consisting of epidemiological data did not indicate any safe BLL under which no adverse effects would occur.

## 3. Assessment of Human Exposure

The WG evaluated the status of exposure to lead in Japan by examining data on estimated exposure from different sources, such as food, water, soil, and dust, as well as data on BLLs measured in Japan.

### 3.1. Exposure from Various Sources

Lead exposure calculated from Japanese total diet studies decreased substantially between 1978 and 1982, as control measures including the regulation of lead in gasoline were implemented in the 1970s. The decline continued during the following decades, and the mean estimated dietary intake of lead decreased from approximately 100 μg/day in 1978 to 9 μg/day in 2019^25^^)^. The contribution of each food category to the overall lead exposure varied according to studies, and no specific food category was identified as a major contributor to lead exposure. This ubiquitous distribution pattern was different from that of methyl mercury or cadmium, for which the food categories to be targeted in risk management were more obvious.

Lead exposure via drinking water is thought to be minor compared with other food categories; the estimated intake from drinking water represented only 2.3% of the total intake in the total diet study of 2019^25^^)^. Apparatus that contain lead in water supply systems have been gradually replaced by lead-free alternatives since the 1990s. The lead concentrations in tap water were below or equal to 0.001 mg/L at more than 95% of the test points in the 2018 survey^26^^)^.

Lead exposure from other sources, such as house dust, soil, and toys, in Japan was estimated using the results of previous studies^27^^-^^29^^)^. Some studies have indicated that exposure from non-dietary sources might be important in children^30^^,^^31^^)^; however, the estimated values of the contribution of non-dietary sources to the overall lead exposure were highly variable depending on the studies and thus no representative value could be derived. The use of different analytical methods for measuring lead concentrations in environmental media and the estimation of lead intake values by applying different ingestion rates were thought to be the source of variance.

### 3.2. Blood Lead Level

BLL is considered to represent recent exposure to lead from various sources, such as food, water, soil, house dust, and polluted air. The BLL is the most common index of lead exposure in epidemiological studies. However, no national population-based survey of BLLs has been conducted in Japan. Thus, the WG collected information on BLLs from recently published studies to estimate the current exposure level of the Japanese people by reviewing the available data, although each study had its own limitations.

**Fig. 1.**
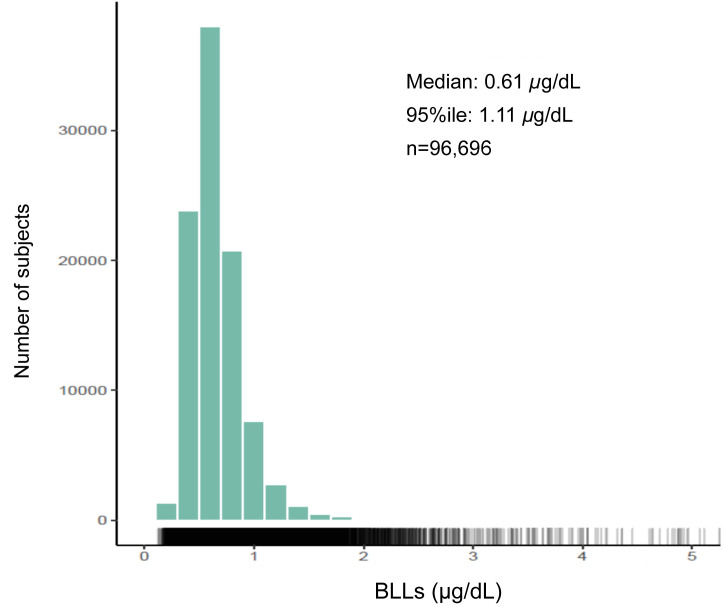
Blood lead levels in subjects (pregnant women) of the Japan Environment and Children’s Study

Regarding adults, only one study included both sexes. This study, conducted by the Ministry of the Environment between 2012 and 2016, reported a median BLL of 1.1 μg/dL for 404 individuals aged between 40 and 59 years^32^^)^. Other studies have included only women; most of which were pregnant women. Among these, the Japan Environment and Children’s Study (JECS) had the largest number of participants and included various regions across the country. The latest data from JECS indicated that the median BLL was 0.61 μg/dL and the 95th percentile BLL was 1.11 μg/dL among 96,696 pregnant women recruited between 2011 and 2014 (Fig. 1)^33^^)^. Compared with BLLs in pregnant women in the 1980s, the current BLLs in pregnant women reported by the JECS were decreased by one fifth to tenth^34^^)^.

However, it might not be appropriate to consider BLLs in pregnant women as representative values of the entire Japanese population, because BLLs vary by age and sex. National surveys in other countries have demonstrated that BLLs increase gradually with age in adults. These surveys also show a clear sex difference in BLLs; BLLs are higher in men than in women^35^^,^^36^^,^^37^^)^. In addition, it is noteworthy that BLLs in pregnant women tend to be lower than those in non-pregnant women of the same age because of an increase in plasma volume during the first and second trimesters of pregnancy. A study based on data from the NHANES 1999–2016 showed that BLLs in pregnant women were slightly lower (about 90%) than those in non-pregnant women of childbearing age^38^^)^.

This study using the NHANES data also reported a geometric mean BLL of 0.62 μg/dL and a 90th percentile BLL of 1.30 μg/dL among 1,283 pregnant women. By comparing the BLLs of pregnant women reported by JECS and those reported by NHANES, the WG considered that the BLLs in the Japanese population were in the same range as those in the American population. As the BLLs in the United States were relatively low in both sexes and across age groups compared to those in other developed countries, the lead exposure level in Japan was estimated to be among the lowest in the world.

**Fig. 2.**
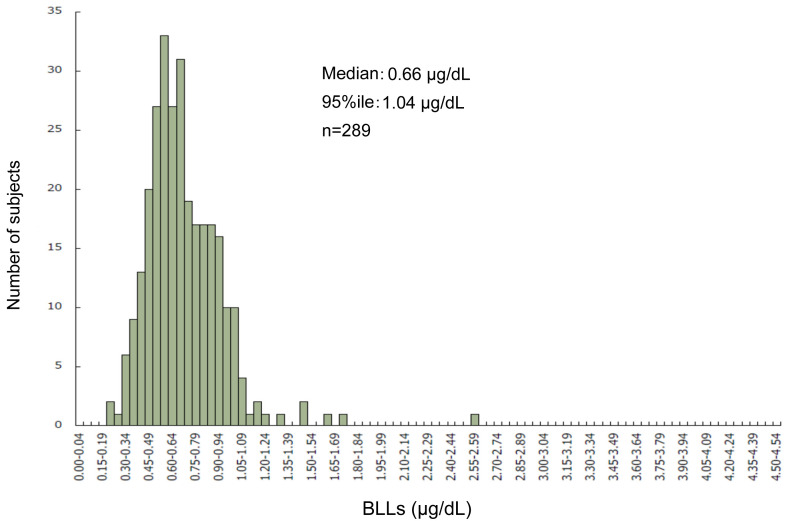
Blood lead levels in subjects (12-year-old children) of the Tohoku Study of Child Development

Data on BLLs in Japanese children are also sparse. Most studies have been conducted in limited geographical areas and with samples of less than 300 participants. Considering the limitations of the studies, the WG decided to use the data of the latest article from the Tohoku Study of Child Development to estimate the current exposure in Japanese children^4^^)^. Accordingly, the estimated median BLL was 0.66 μg/dL and the 95th percentile BLL was 1.04 μg/dL among 289 children (148 boys) aged 12 years old, who were born between 2002 and 2006 (Fig. 2)^33^^)^. However, these values should be interpreted with caution because BLLs are known to rise after infancy, reaching the highest level at around 2 years of age, and then decline slightly during childhood and adolescence, dropping to the lowest level between the ages of 12 and 19 years old^7^^,^^35^^,^^37^^)^. Hence, the BLLs observed among 12-year-old children cannot be considered representative of children of all ages.

Owing to the lack of population-based surveys with representative samples at the national level in Japan, it was difficult to obtain an overall picture of the current status of BLLs for the entire population. Nevertheless, judging from the available data, the WG concluded that the average BLL of the Japanese population was below or equal to 1 μg/dL. The WG also noted exceptionally high BLLs in some study participants, although the sources of exposure were not clarified in the original studies. Further studies are warranted to elucidate the reasons for elevated BLLs in some Japanese individuals.

## 4. Conclusions

After careful consideration of the available evidence, the WG reached the following conclusions: exposure to lead has been substantially reduced in Japan since the implementation of control measures in the 1970s, and the current average BLL of the Japanese population is estimated to be below or equal to 1 μg/dL; the evidence suggesting health effects of low-level lead exposure has accumulated over time, and the existing body of evidence indicates that BLLs in the range of 1–2 μg/dL might be associated with some effects on human health; further, given that the estimated average BLL of the Japanese population is close to the levels at which some effects related to lead exposure are suggested to occur by epidemiological studies, measures to reduce lead exposure are deemed indispensable, and their adequate implementation should be continued.

## 5. Future Perspectives

This risk assessment of lead was conducted based on data available at the time of evaluation. There are many deficiencies in the current scientific literature: lack of values of BLLs that are representative of the entire Japanese population as well as of quality-controlled data on exposure levels from both dietary and non-dietary sources and prospective observational data on the effects of low-level lead exposure in Japan. There are still uncertainties in the present assessment due to the paucity of data. Further research is needed to fill these data gaps and reduce uncertainties in the assessment.

### 5.1. Discussions on Setting a reference Value

There were discussions among the WG members on whether to establish a reference value of BLLs for the Japanese population using the distribution of BLLs reported in recent studies conducted in Japan. Since the WG concluded that it is difficult to identify a BLL that would be health-protective because of the uncertainties associated with the effects of such low levels of lead exposure observed in Japanese individuals, an interesting option could be to define a level of reference simply based on the current exposure status of the Japanese people (e.g., 97.5th percentile of the BLL distribution in the Japanese population). This level of reference can be used to identify individuals with elevated BLLs and trigger public health actions^39^^)^. However, setting this value is within the remit of risk management. Moreover, there are no data on the current BLLs in the Japanese population that can be considered representative of the entire population in terms of sex, age, and region. Therefore, it was decided not to refer to the concept of the reference value in the risk assessment report of the FSCJ. Nonetheless, it might be useful for the risk management body to deliberate on the use of reference values to assess the effectiveness of control measures aimed at reducing human lead exposure.

### 5.2. Conversion of BLLs into Dietary Exposure Values

The WG members also had prudent discussions on whether to conduct the risk assessment based on dietary lead exposure. Since the request from the MHLW was an assessment of the risks to human health related to the presence of lead in food, the evaluation of dietary lead intake values might seem natural. However, human exposure to lead can also arise from non-dietary sources, such as house dust and soil. Environmental sources might be especially important for children because they are more in contact with these environmental media.

Lead in the blood is considered to be the best indicator of overall human exposure from various sources. Since most epidemiological studies on the health effects of lead have been conducted using BLL as an index of lead exposure, the results of the assessment on the health effects of lead are given in BLL. Hence, in the current risk assessment of the FSCJ, the risks that lead poses to the health of Japanese people were evaluated by directly comparing the BLLs associated with some effects on human health and the BLLs recently reported in Japan.

The WG recognized that there were toxicokinetic models that could be used to convert BLLs into dietary exposure values; however, these models were developed during a period when the concentration of lead in the environmental media was high. Thus, it is uncertain whether the parameters used in the models are still valid at present. In addition, data on the concentrations of lead in Japanese environmental media, such as house dust and soil, are limited in sample numbers and geographic distributions. As a result, the representative values of Japanese environmental media to be used for calculation with the models are currently lacking. Considering these uncertainties and limitations, the WG members decided to conduct risk assessment based on levels of lead in the blood and not on values of lead in food estimated by models.

Risk assessment of lead was a rare case for the FSCJ in that it considered risks associated with exposure not only from food but also from other environmental sources, and risks were evaluated based on the concentrations of lead in blood samples. The conclusions without the establishment of reference values for intake from food might seem atypical for a risk assessment conducted by the FSCJ; however, given the ubiquitous occurrence of lead in the environment, it is necessary to assess the risks of human exposure to lead from all sources, including non-dietary sources.

### 5.3. Urgent Call for a scheme of Human Biomonitoring

Through the process of the risk assessment of lead by the FSCJ, many data gaps were identified. Among these gaps, the lack of data on BLLs from representative study samples of the Japanese population is crucial.

Lead in the blood is a good biomarker of overall lead exposure reflecting exposure from all routes, and is a good metric for epidemiological studies assessing lead exposure. Data on BLLs have been accumulated in developed countries over the past decades, often in the framework of human biomonitoring at the national level. Analyses of these data clearly indicate that BLLs significantly decreased with the implementation of control measures in the 1970s to reduce lead exposure, especially via the inhalation route. As shown by this temporal trend of BLLs, human biomonitoring can provide valuable information on the effects of control measures at the population level. To ensure the comparability of data across time, well-designed human biomonitoring programs using standardized methods are indispensable.

Thus, a human biomonitoring program to monitor the lead exposure levels of the Japanese people at the national level is warranted. This kind of human biomonitoring can provide useful information for assessing time trends and geographic patterns of lead exposure in Japan, for identifying any subpopulation with elevated lead levels, and for examining the effectiveness of management measures to reduce lead exposure. Moreover, such a human biomonitoring program could be used to collect data on other substances of public health concern. That is the reason the risk assessment report on lead by the FSCJ was concluded with a sentence underlining the need for a national human biomonitoring program in Japan.
